# *Locus coeruleus* complex of the family *Delphinidae*

**DOI:** 10.1038/s41598-018-23827-z

**Published:** 2018-04-03

**Authors:** Simona Sacchini, Manuel Arbelo, Cristiano Bombardi, Antonio Fernández, Bruno Cozzi, Yara Bernaldo de Quirós, Pedro Herráez

**Affiliations:** 1 Veterinary Histology and Pathology, Institute of Animal Health, University of Las Palmas de Gran Canaria, Veterinary School, Trasmontaña s/n, Arucas, Las Palmas 35416 Spain; 2 Department of Veterinary Medical Science, University of Bologna, Ozzano dell’Emilia, Bologna 40064 Italy; 3 Department of Comparative Biomedicine and Food Science, University of Padova, viale dell’Università 16, Legnaro (PD), 35020 Italy

## Abstract

The *locus coeruleus* (LC) is the largest catecholaminergic nucleus and extensively projects to widespread areas of the brain and spinal cord. The LC is the largest source of noradrenaline in the brain. To date, the only examined *Delphinidae* species for the LC has been a bottlenose dolphin (*Tursiops truncatus*). In our experimental series including different *Delphinidae* species, the LC was composed of five subdivisions: A6d, A6v, A7, A5, and A4. The examined animals had the A4 subdivision, which had not been previously described in the only *Delphinidae* in which this nucleus was investigated. Moreover, the neurons had a large amount of neuromelanin in the interior of their perikarya, making this nucleus highly similar to that of humans and non-human primates. This report also presents the first description of neuromelanin in the cetaceans’ LC complex, as well as in the cetaceans’ brain.

## Introduction

The *locus coeruleus* (LC) is a densely packed cluster of noradrenaline-producing neurons located in the upper part of the rostral rhombencephalon, on the lateral edge of the fourth ventricle. The LC is the largest catecholaminergic nucleus of the brain, and it supplies noradrenaline to the entire central nervous system. Noradrenaline neurons are located in the medulla oblongata and pons (termed A1-A7 divisions), while adrenaline neurons are located only in the medulla oblongata, near A1-A3 (and termed C1-C3)^1^. The LC sends projections throughout the brain, including to the neocortex, thalamus, midbrain, hindbrain, cerebellum, and spinal cord^2^. It represents the only source of noradrenaline for these structures^3^, and specifically for the cerebral, limbic and cerebellar cortices. The LC extends from the caudal limit of the inferior colliculi to the motor nucleus of the trigeminal nerve^4^. The main divisions making up the LC are located in the rostral rhombencephalon: the A4 division, in the lateral part of the roof of the fourth ventricle; the A5 division, medial to the outgoing fibres of the *nucleus facialis* and lateral to the *nucleus olivaris superior*; the A6 division, mainly at the level of the rostral third of the *griseum pontis*; and the A7 division, at the level of the caudal third of the *griseum pontis*^5^. The LC is involved in attention, behavioural activation, and arousal. Moreover, the LC plays a key role in the stress circuit, and clinical evidence suggests a relationship between the central noradrenergic system and conditions of fear and anxiety^6^. The LC is of great interest for its involvement in neuronal loss in Alzheimer’s disease (AD) and Parkinson’s disease (PD). In later life, lower LC neural density is associated with slower responses to alerting stimuli^7^ and eventually with cognitive decline^8^.

Notwithstanding the attention received in recent decades, there are still unresolved issues regarding the brain of cetaceans. One of the primary difficulties is obtaining fresh brain samples from such unique animals with large brains. The only hitherto examined cetacean species for LC have been the bottlenose dolphin^9^, the harbour porpoise (*Phocoena phocoena*)^10^, and the minke whale (*Balaenoptera acutorostrata*)^11^.

## Results

### LC extension in the family *Delphinidae*

The LC of the examined species was a bilateral brain structure, of a considerable size, especially in its rostro-caudal extension, confirmed by Tyrosine Hydroxylase (TH)-immunoreactivity. TH is the first enzyme involved in catecholamine biosynthesis and catalyses the conversion of L-tyrosine to L-DOPA. For this reason, it is considered the selective neuromarker for nuclei that synthesize catecholamines^12–17^, including the LC^9,13,18^.

**Figure 1:**
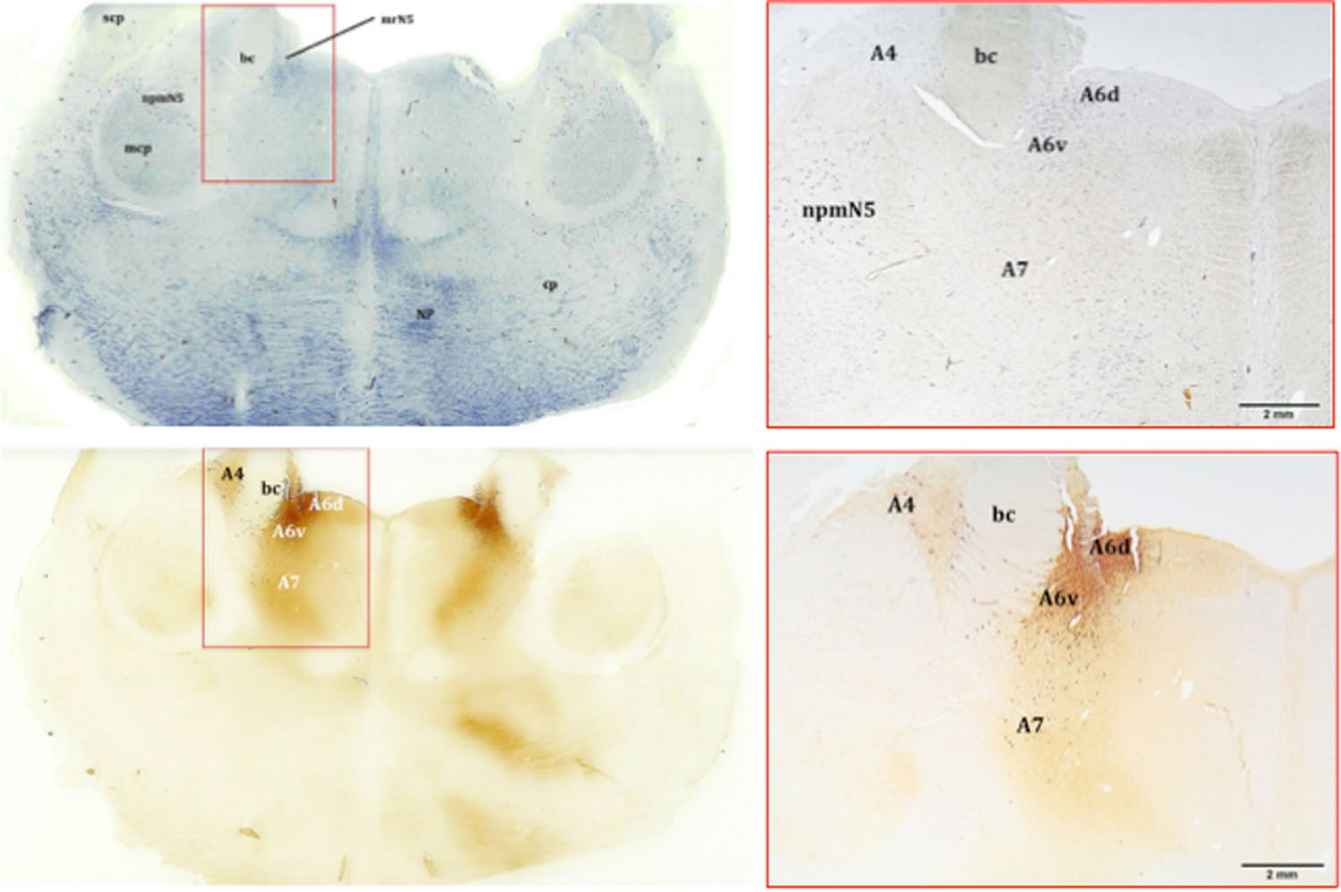
Coronal section of the brainstem. Localization of the A4, A6, and A7 divisions. Thionine staining (upper pictures) and distribution of the TH immunoreactive neurons which make up the LC complex, free-floating immunolabelling (lower pictures). Atlantic spotted dolphin, *Stenella frontalis*. bc, *brachium conjunctivum*; npmN5, mesencephalic nucleus of the trigeminal nerve; A6d, dorsal; A6v, ventral.

**Figure 2:**
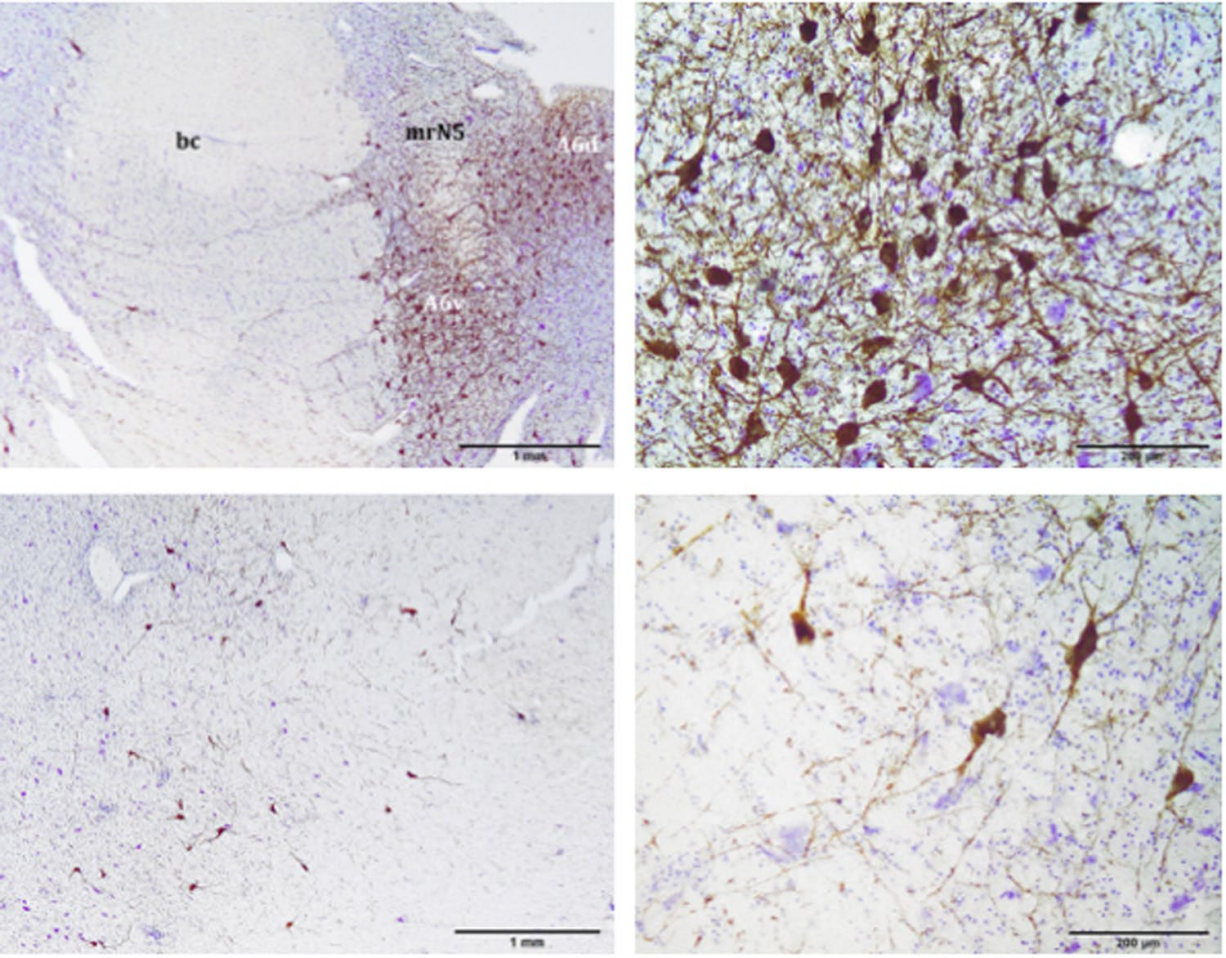
A6d and A6v subdivisions (upper pictures) and A7 division (lower pictures). A6d is entirely restricted to the PAG and medial to the *bc*, while A6v is located just outside the PAG, ventro-lateral to the A6d and medial to the *bc*. A6d and A6v are separated by bundles of nerve fibres belonging to mrN5. A7 is located in the pontine tegmentum, ventral to A6. Atlantic spotted dolphin, *Stenella frontalis*. TH free-floating immunolabelling, counterstained with thionine. bc, *brachium conjunctivum*; mrN5, mesencephalic root of the trigeminal nerve.

**Figure 3:**
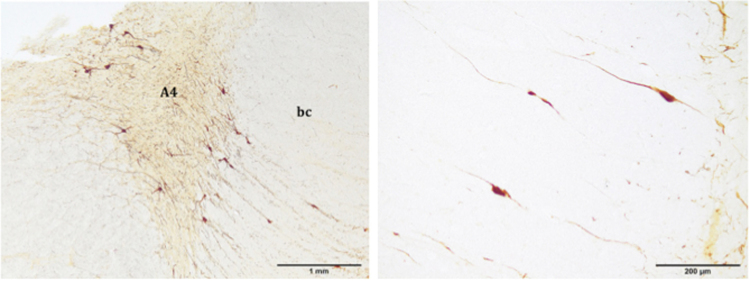
Distribution of the TH immunoreactive neurons, which make up the A4 division. The A4 division is located in the lateral recess of the fourth ventricle, lateral to the *bc*, and shows a triangular morphology. The A4 divison has two portions, one lateral and one medial (right picture). Atlantic spotted dolphin, *Stenella frontalis*. TH free-floating immunolabelling.

**Figure 4:**
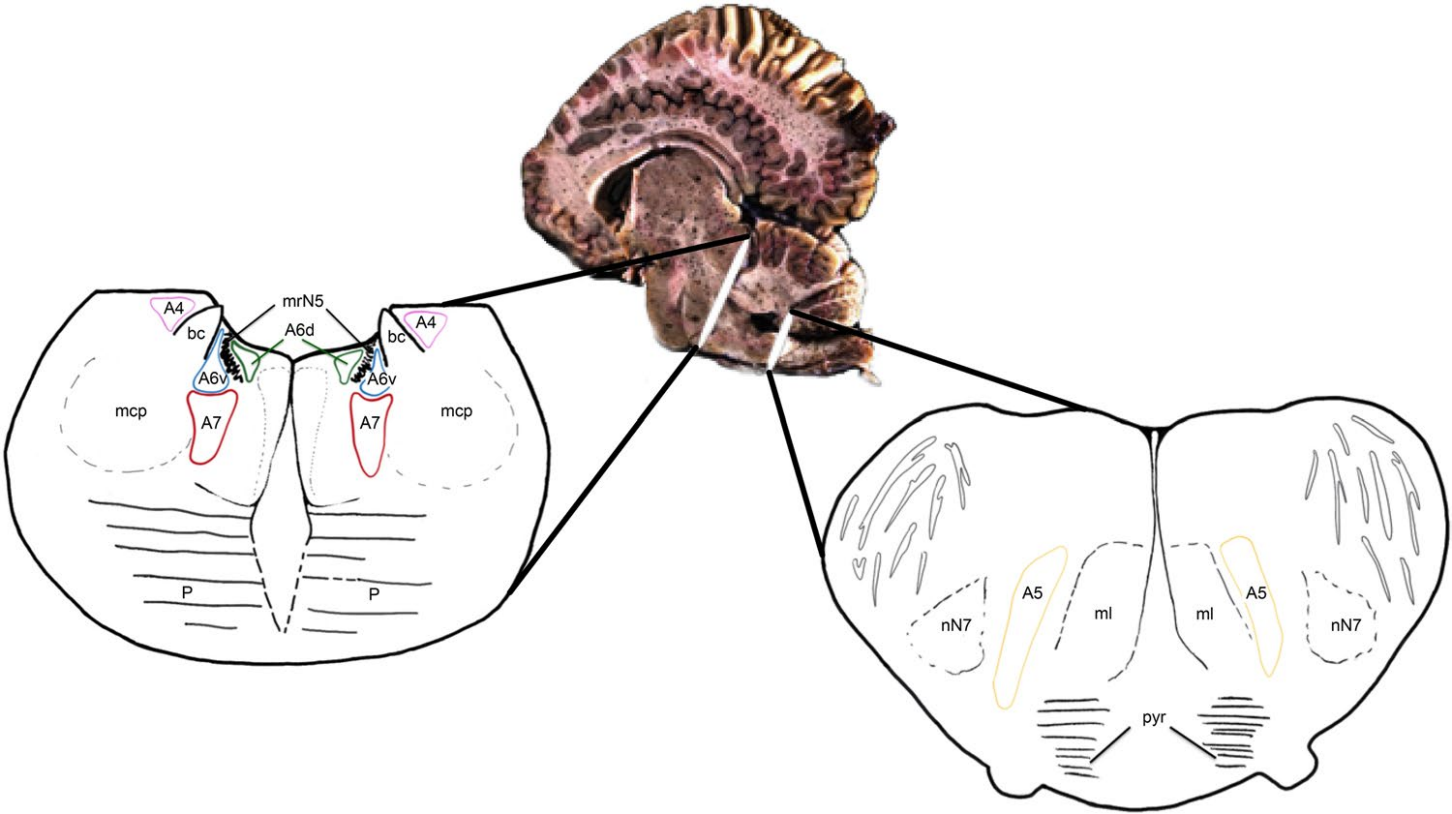
Schematic representation of the divisions and subdivisions making up the LC complex of the family *Delphinidae*. Coronal section of the metencephalon (left picture) and the myelencephalon (right picture). bc, *brachium conjunctivum*; mcp, middle cerebellar peduncle; ml, medial lemniscus; mrN5, mesencephalic root of the trigeminal nerve; nN7, nucleus of the facial neve; P, pons; scp, superior cerebellar peduncle; pyr, pyramids.

The LC complex consisted of four divisions (A4, A5, A6, A7), which occupied both the periaqueductal grey (PAG) and the pontine tegmentum (Figs 1–3 and 4). At the caudal most level of the inferior colliculi, caudal to the commissure of the inferior colliculi, the motor nucleus of the trochlear nerve (mnN4) marked the rostral limit of the LC. The mnN4 was a bilateral group of multipolar neurons, evenly distributed in a circle and located in the PAG. The perikarya were large, polygonal, and intensely stained with thionine. At this point, LC neurons were not evident, confirmed by the lack of TH-immunoreactivity. Caudal to the mnN4, just when it came to its end, the first LC neurons appeared, occupying the PAG. Very few neurons exclusively occupying the PAG were labeled by TH-immunostaining.

The LC developed around a bilateral structure with a remarkable horn shape in cross sections: the *brachium conjunctivum* (*bc*) of the superior cerebellar peduncles, formed by nerve fibres connecting the brainstem to the cerebellum (Figs 1–3 and 4).

Jointly with the *bc*, the LC began to acquire a remarkable development, including the A6d (dorsal) and the A6v (ventral) subdivisions, separated by the mesencephalic root of the trigeminal nerve (mrN5) (Fig. 2, upper pictures). In this structure, elongated polygonal neurons were found, belonging to the A6v subdivision. Ventral to the A6, the A7 division was present. The A7 was made up of large, scattered neurons (Fig. 2, lower pictures).

Rostrally, the LC of the examined species was reduced in size, with less neuronal density, but its size increased caudally, reaching a maximum size in the metencephalic region. Entering the metencephalic region, the A4 division of the LC was likely (Fig. 3). The A4 division was latero-dorsal to the *bc* and medial to the mesencephalic nucleus of the trigeminal nerve (npmN5) (Figs 1, 3 and 4), acquiring a triangular shape and having a few neurons with a polygonal morphology (Fig. 3). Some of its neurons, with a smaller size and a fusiform morphology, invaded the *bc*, constituting a medial subdivision of the A4 division (Fig. 3, right picture). Caudally, the LC increased its neuronal density and clearly grouped in three of its four divisions (A4, A6, and A7) (Figs 1 and 4, left picture). The benchmark of the ending of the LC was the motor nucleus of the trigeminal nerve. Nevertheless, the A5 division appeared caudal to it (Fig. 4, right picture). The reference structures used as a guide in the identification of the A5 division of the examined species were the ventral cochlear nuclei (VCN), structures of great development in the examined species and therefore easily identifiable. VCNs were located in the ventro-lateral tegmentum, dorsal to the trapezoidal body, at the outlet of the vestibulocochlear nerves. The A5 division was limited to a very specific region, unlike the other divisions. Caudal to the VCNs, A5 neurons were medial to the nucleus of the facial nerve (nN7). Moreover, the A5 division was lateral to the medial lemniscus (ml), the medial reticular formation (mrf) and the pyramids (pyr) (Fig. 4, right picture). The A5 division had an elongated morphology dorso-ventrally, with a very low neuronal density (Fig. 4, right picture). The last neurons belonging to the A5 division were located at the beginning of the inferior olivary complex.

Thus, in the present study, the LC extended from the caudal limit of the motor nucleus of the trochlear nerve to the rostral limit of the motor nucleus of the trigeminal nerve. The LC had four divisions (A4, A5, A6, and A7), which corresponded to five major subdivisions (A4, A5, A6d, A6v, and A7) (Figs 1–3 and 4). The A6d subdivision was a group of densely packed neurons, entirely restricted to the PAG and medial to the *bc* (Fig. 1; 2, upper pictures; 4, left picture). The A6v subdivision was located just outside the PAG, ventro-lateral to A6d and medial to the *bc*, even occupying the *bc* with few neurons (Figs 1; 2, upper pictures; 4, left picture). The A6d and A6v subdivisions were separated by bundles of nerve fibres belonging to mrN5. A6v neurons were distributed in a less compact manner than the A6d neurons (Fig. 1; 2, upper pictures; 4, left picture). The A7 division was located in the pontine tegmentum, ventral to the A6 division and expanding the A6v subdivision (Fig. 1; 2, lower pictures; 4, left picture). Its neurons occupied the ventral tegmentum and were distributed in a more widespread manner (Fig. 2, lower pictures) compared to the previous two subdivisions. The A4 division was the lateral-most and dorsal-most division, in proximity to the superior cerebellar peduncle and lateral to the *bc* (Figs 1, 3 and 4, left picture). The A5 division was confined to the caudo-ventral tegmental area between the VCNs and the inferior olivary complex (Fig. 4, right picture).

### Cytoarchitecture of the LC

**Figure 5:**
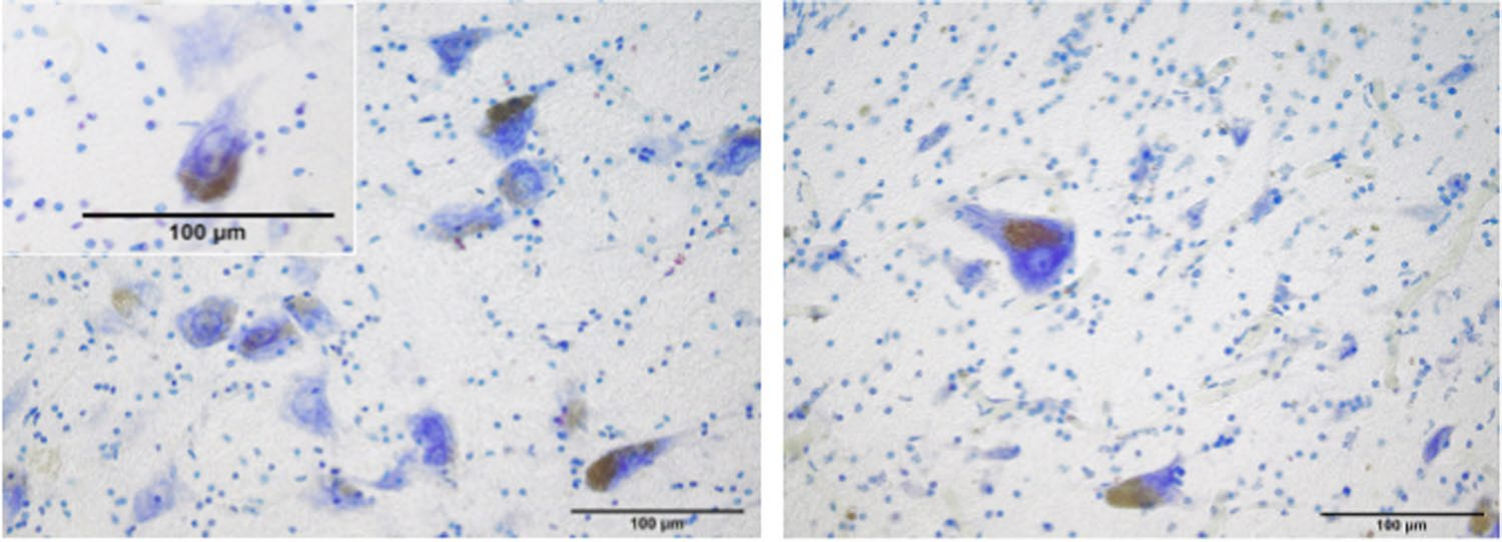
Neuromelanin in the cytoplasm of the LC’ perikarya. Most neurons have the typical dark brown-blackish neuromelanin granules, occupying either a pole of the cell or even half of the cytoplasm. The nucleus is placed in a central position with an evident nucleolus. Around the nucleus, Nissl granules are distributed homogeneously. Atlantic spotted dolphin, *Stenella frontalis* (left) and Risso’s dolphin, *Grampus griseus* (right). Thionine staining.

LC neurons showed a high affinity to thionine both in the soma and in the axon hillock (Fig. 5). Neurons had a polygonal cell body with 3 or 4 sides. The nucleus was placed in a central position with an evident nucleolus. Around the nucleus, Nissl granules were distributed homogeneously (Fig. 5).

In the five divisions, the neuronal morphology and area were very similar, with a variable size between 500 and 1,400 µm^2^ and a perimeter between 100 and 180 µm (Table 1). Only the neurons with an evident nucleus were included in the perikaryal area analysis. Most neurons, although not all of them, had typical dark brown to blackish neuromelanin granules (NM) within the cytoplasm (Fig. 5). Neuromelanin granules occupied either a pole of the cell or even half of the cytoplasm. In the latter case, the granules had a darker aspect (Fig. 5).

**Table 1:** Perikaryal areas of the neurons in the LC of the family *Delphinidae*.

LC subdivision	Mean Area ± SD µm^2^	Min. Area µm^2^	Max. Area µm^2^
A6d	845,18 ± 148,82	575,688	1,224.204
A6v	837,76 ± 191,99	370,2957	1,740.0979
A7	1,062.54 ± 205,34	553,0882	1,935.5197
A4	795,9 ± 176,19	608,6918	1,110.7137
A5	799, 41 ± 174, 54	453,7589	1,156.9248

### Neuronal Tyrosine Hydroxylase immunoreactivity in the LC

**Figure 6:**
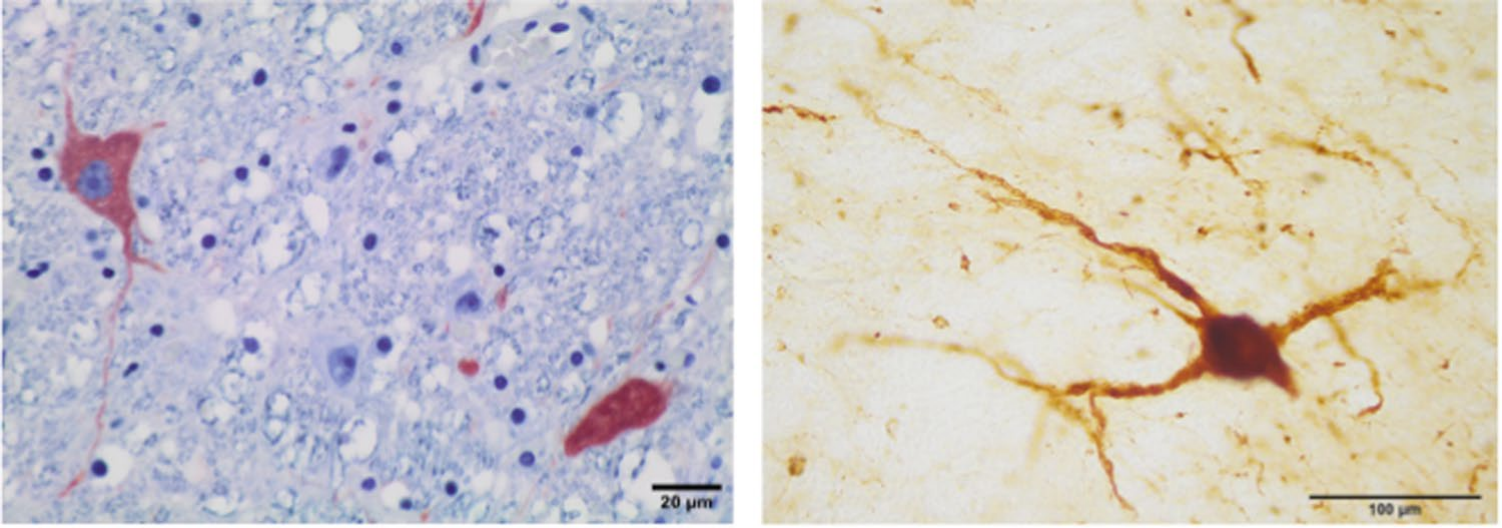
TH immunoreactive neurons of the A6d subdvision from a paraffin embedded sample (left). Medium and large size neurons show a polygonal morphology with 3 or 4 sides and 3 or 4 primary neurites. Bottlenose dolphin, *Tursiops truncatus*. TH immunolabelling, counterstained with a non-commercial Mayer’s haemalum solution; TH immunoreactive neuron of the A5 divsion (right). Atlantic spotted dolphin, *Stenella frontalis*. TH free-floating immunolabelling.

By TH immunostaining, the LC and its stained neurons were clearly recognizable (Fig. 1, lower pictures). Likewise, in the neuropil a dense neurite interlace was intensely stained (Figs 2 and 3). Medium and large sized neurons had a polygonal morphology with 3 or 4 sides, 3 or 4 primary neurites, 1 or 2 secondary, and 1 or 2 tertiary dendrites (Fig. 6).

In the five divisions, the neuronal morphology and area were very similar, with a variable size between 500 and 1,400 µm^2^ and a perimeter between 100 and 180 µm (Table 2).

**Table 2:** Perikaryal areas of the TH-ir neurons in the LC of the family *Delphinidae*.

LC subdivision	Mean Area ± SD µm^2^	Min. Area µm^2^	Max. Area µm^2^
A6d	885,6 ± 167,18	394,0804	975,8085
A6v	896,07 ± 195,71	453,73	1425,6081
A7	1072,22 ± 277,28	663,6596	3229,5172
A4	1439,98 ± 291,19	462,6312	1798,0713
A5	587,38 ± 172,43	500,6636	1322,4929

### Stereological analysis

Stereological estimation of the total number of neurons revealed that there were approximately 97,504 TH-immunopositive as well as thionine stained neurons in the LC of the spotted dolphin.

## Discussion

### LC and the A4 division

In our experimental series, the LC of the toothed whales was of considerable size, in terms of length and extension along the brainstem (Figs 1 and 4). This nucleus had a high neuronal density, noticeable even macroscopically by the presence of neuromelanin in the cytoplasm of neurons, which generally gives the LC its typical greyish colour. Stereological count in the spotted dolphin revealed a total number of 97,504 neurons. The number is next to the total amount TH-immunopositive neurons (122,878) observed in the LC of the harbour porpoise^10^. This number is double the number of neurons observed in the human LC (45-46,000 neurons)^4,19^.

In our sections, the extension of the LC in the bottlenose dolphin covered an area very similar to that previously observed in the same species^9^. Previous studies have established that, in general, animals of different orders have different divisions of the LC, while species of the same order have the same subdivisions^9^. Based on the descriptions of the eighties, the LC of the rat consists of four main divisions: A4, A5, A6, and A7^12^. Other studies performed reported that the A4 division was not present in the bottlenose dolphin^9^. According to the latter study, the complexity of the nucleus and its subdivisions was not proportional to the increase in brain size of the dolphin, but, on the contrary, the LC showed a simplified structure. On the other hand, in the harbour porpoise^10^, the LC is made of five distinct divisions: the subcoeruleus diffuse (A7d), within the parvocellular portion of the pontine tegmentum, encircling the ventral aspect of the superior cerebellar peduncle; the subcoeruleus compact (A7sc), forming a continuous nucleus with A7d; the diffuse portion of the LC (called A6d), in the ventrolateral aspect of the pontine periventricular grey matter; the fifth arcuate nucleus (A5), ventral to the trigeminal motor nucleus and medial to the descending limb of the facial nerve; and the dorsomedial division (A4), in the dorsolateral part of the periventricular grey matter, medial to the dorsal aspect of the superior cerebella^10^. In the minke whale, the LC is subdivided into four subdivisions that include the A7d, throughout the lateral and ventral pontine tegmentum anterior to the trigeminal motor nucleus and extending around the superior cerebellar peduncle; the A7sc, within the dorsal pontine tegmentum, immediately adjacent to the A6d nucleus (the diffuse portion of the LC) in the ventrolateral periventricular grey matter; and the A5, medial to the trigeminal sensory and motor nuclei^11^.

The LC’ parcellation of the analysed toothed whales fits with the one used in the bottlenose dolphin^9^, while some differences have been raised drawing a comparison with the one used in the harbour porpoise’ LC^10^. The A6c (compact) of the harbour porpoises stands for the A6d, the A7sc (subcoeruleus compact) stands for A6v, and the A7 diffuse for A7. These differences as well as the huge diversity in the hitherto used LC’ nomenclature, should force the scientific community to shape and standardize an accepted and universal nomenclature for the LC’ parcellation.

In the literature, the small and inconstantly observed A4 division is considered a constituent part of the LC^12,17,20,21^, representing a dorso-lateral extension of the LC^22^. The A4 division has been described in humans^18^, in non-human primates^20^, and in other species such as the cat^23^, the rat, and some birds^24^. However, the A4 division is absent in eutherian mammals, including terrestrial Cetartiodactyla (giraffe^15^, sheep^25^, and river hippopotamus^26^), the elephant^17^, and in monotremes^13^. In all of the examined cetaceans, including the bottlenose dolphin, the A4 division was present. The A4 division was dorsolateral to the *bc*, while A6 and A7 were medial to the *bc*. Similar to that observed in nonhuman primates^20^, the A4 division was located in the lateral recess of the fourth ventricle. Exactly, it was found lateral to the *bc* and the mesencephalic nucleus of the trigeminal nerve (npmN5), having a triangular morphology (Figs 1, 3 and 4). As in some primates, the A4 division was located more caudally than the A6 division^20^. Still, both divisions were present in much of the extension of the LC, unlike in the brown capuchin monkey (*Cebus apella*), where the A4 division appeared at the end of the A6. Similar results were observed in the black-capped squirrel monkey (*Saimiri boliviensis*)^27^, whose A4 division coexisted next to the A6, as in the examined toothed whales. However, in the dog^28^ and in the cat^29^, the A6 and A4 divisions had no defined limits, unlike what was observed in the examined toothed whales, where the separation between the two divisions was evident by the distribution and morphology of the corresponding neurons. Furthermore, similarly to the capuchin monkey^20^, the A4 division of the analysed toothed whales had two portions, one lateral and one medial (Fig. 3). The lateral division was described in the primates as the largest, while the medial division appeared less developed with smaller neurons. In the analysed cetaceans, the lateral subdivision was composed of a few scattered neurons with polygonal morphology (Fig. 3, left picture). The medial part invaded the *bc*, with its smaller size and polygonal neurons, having a more fusiform morphology and a flatter profile (Fig. 3, right picture). Finally, the A4 division may have differences in location depending on the species, caudally and at the level of the end of the A6 division as in the capuchin monkey^20^, or in a more rostral location, coexisting with the A6 division, as in the case of the squirrel monkey^27^. In the capuchin monkey^20^ an A5 division was not described, while the description of the A6 division agreed with the analysed toothed whales. In this primate, the A6 rostral limit was the output of the trochlear nerve, while in the toothed whales, the rostral limit was set by the mnN4. The location was similar, with the A6 division expanding around the mrN5. In the toothed whales, the A6d subdivision was dorso-medial to the mrN5, while the A6v subdivision was ventrolateral to the mrN5. The A6 division, as in primates, did not show a clear separation with the A7 division^20^.

The TH-immunoreactivity confirmed the presence of an A4 division, resembling the same position in humans and non-human primates^4^. The immunohistochemical detection of TH, dopamine beta-hydroxylase (DβH), and phenylethanolamine N-methyltransferase (PNMT) are used to distinguish between uptake and biosynthesis of catecholamines. DβH and TH immunoreactivity along with no PNMT immunodetection, confirm noradrenaline production. Even if catecholamines are similar in their structure and immunohistochemically detectable by TH^1^, their distribution in the brain is quite different. Noradrenaline neurons are located in the medulla oblongata and pons (A1-A7), while adrenaline neurons are placed only in the medulla oblongata. It is also true that neuromelanin is a polymer formed by the oxidative polymerization of dopamine and noradrenaline, and the area where LC is usually detected, does not usually show dopaminergic neurons. Even if TH has been used over time as a good marker for LC neurolocalization^30^, we can only assume that the LC of toothed whales is putative noradrenergic.

In conclusion, the LC complex of the examined toothed whales showed a considerable size compared to other mammals including humans, due to its rostro-caudal extension, the presence of all five subdivisions (A4, A5, A6d, A6v, and A7), and the corresponding size of each of the subdivisions. Some of the 5 subdivisions occupied different areas distant between them. This could be due to the potentially wider area of projection of the catecholaminergic fibres in brains of larger mass. The A6d and the A6v subdivisions, the closest to the fourth ventricle, could reach rostral structures, sending projections mainly to the cortex, passing through the posterior commissure. TH-positive fibres, probably belonging to the LC, have been observed in the large posterior commissure^9,31,32^. The A4 division, the lateral- and dorsal- most division, controls the cerebellum, sending projections through the *bc* and the superior cerebellar peduncle. The A4 projections have been described as directed towards the flocculus and paraflocculus of the cerebellum, with some processes entering the ependyma and others ending subependymally^20^. The A7 and A5 divisions, located in the metencephalic tegmentum, would innervate all the brainstem, and the A5 probably innervates the medulla oblongata and the spinal cord^33^. All of these projections would ensure a noradrenergic neuro-modulatory control by the LC to the entire central nervous system, ensuring a constant vigil for muscle activity in these marine mammals, totally adapted to the aquatic medium. Different studies in terrestrial mammals indicate that the activity of the LC is reduced during the deeper stages of slow-wave sleep and REM sleep^2,34^. It has been suggested that neurons of the LC fire continuously in cetaceans^9^, although the discharge frequency may vary between the left and the right hemisphere^35^. The continuous discharge would enable the maintenance of muscle tone during sleep^32,36^. Behaviours associated with rest periods, such as constant swimming, slow swimming, surface floating, or sleeping on the seabed, are common behaviours reported in many cetaceans, both in captivity and in the wild^32^.

### LC and the Neuromelanin

The study of the LC highlighted the existence of a large amount of neuromelanin within LC neurons (LC-NM). The NM is a polymer formed by the oxidative polymerization of dopamine and noradrenaline, produced by specific brain catecholaminergic populations^36^. Like peripheral melanins, NM has a melanic portion not only composed of eumelanin and pheomelanin residues but is also characterized by the presence of lipids and peptides covalently bound to the melanic component^37^. The NM has not been previously observed in any cetacean’s catecholaminergic area of the brain^9–11,31^. The human brain has the largest amount of NM. A lesser amount is found in the brains of non-human primates and Perissodactyla^38^, and it is absent in other mammals^36^, including laboratory rodents. This suggests that the production of NM is not an inevitable consequence of the production of catecholamines^39^. Only 65% of the noradrenaline- producing neurons in the human brain have NM inside their perikarya^40^. Recent studies have suggested that NM synthesis could play a protective role in the neuron, preventing the accumulation of toxic derivatives of catecholamine synthesis by incorporating the polymer. Another hypothesized function is chemoprotection against harmful substances such as pesticides and other toxic compounds^41^, neuroleptics, ligands, or metals, especially potentially toxic cations such as mercury^42^. Conversely, even more recent studies claim that the release of NM by neurons undergoing necrosis could contribute to the activation of microglia, triggering the neuro-inflammatory process which characterizes the PD^37,43^.

In AD and PD, there could be a loss of neurons in the *susbtantia nigra* and in the LC^36,44^. The presence of NM is a basic feature of vulnerable cells in neurodegenerative processes and suggests an important role of this pigment. The LC-NM has been observed in all the examined toothed whales, except for the bottlenose newborn. This fact represents a striking difference with the situation of the closely related terrestrial Cetardiodactyla^45^, which show no neuromelanin granules in either the *substantia nigra* or the LC. Cetaceans are homeotherms, long-lived species and are located at the top of the marine food chain. Long-lived animals possess characteristics in their brains reflecting an increased longevity, such as the presence of pigments, including the LC-NM. The animals included in this study were adults, juveniles and a newborn. Adults had the greatest amount of LC-NM, while the newborn did not have LC-NM. Unlike skin or retinal melanin, the LC-NM is not present during foetal development and accumulates over decades of life^36^. LC-NM starts to be evident in children at 2–3 months of life, and between 3 and 8 months it increases progressively, being only 30% of the LC-NM in the adult brain^46^. This study has not included neither the quantification in relation to the age of the animal nor the composition of the LC-NM, and future studies should attempt to link the presence of this pigment with potential neurodegenerative processes in cetaceans.

### Concluding remarks

The presence of the A4 subdivision in the LC is exclusive to certain animals, including humans and non-human primates. Moreover, the existence of LC-NM may raise the following question: why does the brain of these animals have certain features in common with the human brain, including from a “neuropathological” point of view? The answer should probably be sought in the lifespan of these animals, which approximates, in many species, the human lifespan. Moreover, cetaceans are long-lived top predators, which are at a high risk from bioaccumulation and biomagnification of a variety of organic and metallic pollutants. Neurodegenerative diseases, such as AD and PD, may be investigated in these animals, focusing attention on the structures that mostly affect humans, including the LC and the *substantia nigra*, among others.

Certain neurodegenerative disease, and/or some aspects of neurodegenerative diseases (AD, PD, and/or other tauopathies and alpha-synucleinopathies) might be investigated in cetaceans, particularly odontocetes^47^. In some species of cetaceans, deposits of ß-amyloid peptide have been observed throughout the brain, including the cerebellum and the medulla oblongata, where such deposits have only been reported in the more severe AD cases^47^. Moreover, it was demonstrated that the Aß1-42 peptide was 100% identical to the human peptide in three dolphin species (*Grampus griseus*, *Stenella coeruleoalba*, and *Tursiops truncatus*)^47^. Similar data have also been recently described in the same species. Amyloid plaques and tau fibrillary tangles have been observed as diffuse deposits in the parietal cortex and more compact senile plaques in the cerebellum^48^.

## Materials and Methods

From 2011 to 2016, 281 cetaceans stranded and died in the Canary Islands (Spain) for unknown reasons. Systematic pathological studies were performed on the carcasses to determine the cause of death and/or stranding. Required permission for the management of stranded cetaceans was issued by the environmental department of the Canary Islands’ Government and the Spanish Ministry of Environment. No experiments were performed on live animals.

Brains were obtained from seventeen specimens of six different species of the family *Delphinidae* of the suborder Odontoceti: short-finned pilot whale (*Globicephala macrorhynchus*) (n = 1), Risso’s dolphin (*Grampus griseu*s) (n = 2), striped dolphin (*Stenella coeruleoalba*) (n = 4), Atlantic spotted dolphin (*Stenella frontalis*) (n = 2), common dolphin (*Delphinus delphis*) (n = 3), and bottlenose dolphin (*Tursiops truncatus*) (n = 5). The animals stranded in the Canary Islands between 2011–2016, apart from three bottlenose dolphins, one newborn and two adults, come from a controlled environment and died of natural causes.

Brains were removed, coded for freshness and dissected within 24 hours after death. The postmortem times were different but never exceeded 48 hours. Brains were immersion-fixed at the time of necropsy in 4% formaldehyde in phosphate-buffered saline (PBS; pH 7.4). Before immersion, some longitudinal cuts (2 to 4) were made in both the cerebral and cerebellar hemispheres. In each cerebral and cerebellar hemisphere, at least one of the cuts exposed the lateral ventricles and allowed the fixative to enter the ventricular system. To respect the optimum volume ratio of tissue fixative, which is 10:1^49^, brains were immersed in a container with a minimum volume of 20 litres of fixative. The brains were left in the fixative for at least 72 hours. Coronal sections were produced with a slicing machine. Sections (1–2 cm thick) were returned to the fixative for at least 48 additional hours before proceeding to sampling. Brainstem samples were postfixed in 4% buffered formalin. After rinsing in PBS, samples were cryoprotected in 30% sucrose solution in PBS (pH 7.4) at 4 °C to avoid freezing artefacts. Serial, rostro-caudal, 50 µm-thick coronal sections of the rhombencephalon (from the caudal-most level of the inferior colliculi to the rostral myelencephalon) were made using a sliding freezing microtome. Every fourth serial section was collected for thionine staining, and the adjacent sections were used for immunohistochemistry. The sections were stored in PBS (pH 7.4) containing sodium azide (0.01%).

Of the seventeen animals, eleven specimens were added later to the study, and the fixed brain samples were dehydrated through a series of graded ethanol baths and then infiltrated with paraffin wax. The tissues were then embedded into paraffin wax blocks.

### Thionine staining

Sections adjacent to immunoperoxidase were stained with thionine. It is a common nuclear stain and can be used for the demonstration of Nissl substances in neurons. Sections were taken out of the PBS-containing sodium azide, mounted on gelatin-coated slides, and dried. Sections were defatted, soaking the sections for 1 hour in a mixture of chloroform and 100% ethanol (1:1). Sections were later rehydrated through a graded series of 100%, 96%, 80%, 70%, and 50% ethanol and dH_2_O (2 minutes each), stained 30 minutes in a 0.125% thionine (Fisher Scientific) solution, dehydrated and coverslipped with Entellan.

### Immunoperoxidase staining

Part of the immunoperoxidase staining procedure was carried out on free-floating sections and using a standard immunohistochemical ABC protocol for paraffin embedded samples. Sections proceeding from formalin fixed free-floating sections were treated with 3% H_2_O_2_ in PBS for 30 minutes at room temperature (RT) to eliminate endogenous peroxidase activity and rinsed in PBS (three times, 10 minutes each). To block non-specific binding, sections were incubated in a solution containing 10% normal horse serum (Vector, S-2000), containing 0.5% Triton X-100 (Merck, Darmstadt) to permeabilize the tissue, in PBS for 2 hours at RT. Thereafter, a first set of sections was incubated in a monoclonal Anti-Human Tyrosine Hydroxylase (Monosan, MONX10786), Clone 1B5, diluted 1:200, overnight at 4 °C. The accuracy of the antibody was confirmed by immunolabeling in the dopaminergic *substantia nigra* neurons and lack of immunostaining in some no catecholaminergic areas (cortex, amygdaloid complex, and other no catecholaminergic brainstem nuclei).

The following day, the sections were rinsed in PBS (three times, 10 minutes each) and incubated for 45 minutes at RT with a secondary antibody, biotinylated horse anti-mouse (Vector Laboratories, BA-2000, diluted 1:200), in a solution containing 1% normal horse serum in PBS. The sections were rinsed in PBS and incubated with an avidin-biotin complex (ABC, Vector Laboratories; PK-4000) for 1 hour at RT. Finally, the sections were developed using a 3,3′-diaminobenzidine (DAB) peroxidase kit (Vector Laboratories, SK-4100). The sections were mounted on coated slides and dried overnight. Slides were then dehydrated in ethanol, cleared in xylene and coverslipped with Entellan (Merck, Darmstaldt, Germany). In addition, some TH immunolabeled sections were counter-stained using thionine prior to dehydration and coverslipping.

On the other side, paraffin embedded samples were deparaffinated with xylene and rehydrated in graded ethanol. Immunohistochemistry was carried out using the standard immunohistochemical ABC protocol. Endogenous peroxidase activity was blocked by incubation with 3% H_2_O_2_ in methanol for 30 minutes at RT. Antigen retrieval was performed by the wet autoclave method of Shin^50^ at 118 °C for 5 minutes. The primary antibody (diluted 1:50) was incubated on sections overnight at 4 °C. Sections were developed using the DAB peroxidase kit or the 3-amino-9-ethylcarbazole (AEC) peroxidase substrate kit (Vector Laboratories, SK-4200). Sections were counter-stained using thionine prior to dehydration and coverslipping (DAB labelling) with a non-commercial Mayer’s haemalum solution (AEC labelling).

### Stereological analysis

The numbers of TH-immunopositive and thionine stained neurons in the LC were manually determined using the Digital software Imaging Solutions, CellA. An OLYMPUS BX41 light microscope was used for the counts. The number of neurons was measured at every third slide. A complete count of the total number of the neurons of the LC was possible only in the spotted dolphin. Due to complementary neuropathological examinations performed in our department, only the spotted dolphin brainstem was entirely used for neuroanatomical studies, while the other brainstems were also used, both fresh and fixed samples, for additional investigations.

### Data availability statement

We have provided in the manuscript all of the necessary data to support our results.
